# X-ray Micro-Computed Tomography: An Emerging Technology to Analyze Vascular Calcification in Animal Models

**DOI:** 10.3390/ijms21124538

**Published:** 2020-06-25

**Authors:** Samantha J. Borland, Julia Behnsen, Nick Ashton, Sheila E. Francis, Keith Brennan, Michael J. Sherratt, Philip J. Withers, Ann E. Canfield

**Affiliations:** 1 Division of Cardiovascular Sciences, School of Medical Sciences, Faculty of Biology, Medicine and Health, University of Manchester, Manchester M13 9PL, UK; nick.ashton@manchester.ac.uk (N.A.); ann.canfield@manchester.ac.uk (A.E.C.); 2 Henry Royce Institute, Department of Materials, School of Natural Sciences, Faculty of Science and Engineering, University of Manchester, Manchester M13 9PL, UK; julia.behnsen@manchester.ac.uk (J.B.); p.j.withers@manchester.ac.uk (P.J.W.); 3 Department of Infection, Immunity and Cardiovascular Disease, Medical School, University of Sheffield, Sheffield S10 2RX, UK; s.francis@sheffield.ac.uk; 4 Division of Molecular & Clinical Cancer Studies, School of Medical Sciences, Faculty of Biology, Medicine and Health, University of Manchester, Manchester M13 9PL, UK; keith.brennan@manchester.ac.uk; 5 Division of Cell Matrix Biology & Regenerative Medicine, School of Biological Sciences, Faculty of Biology, Medicine and Health, University of Manchester, Manchester M13 9PL, UK; michael.sherratt@manchester.ac.uk

**Keywords:** vascular calcification, micro-CT, mouse models, histology, correlative microscopy

## Abstract

Vascular calcification describes the formation of mineralized tissue within the blood vessel wall, and it is highly associated with increased cardiovascular morbidity and mortality in patients with chronic kidney disease, diabetes, and atherosclerosis. In this article, we briefly review different rodent models used to study vascular calcification in vivo, and critically assess the strengths and weaknesses of the current techniques used to analyze and quantify calcification in these models, namely 2-D histology and the *o*-cresolphthalein assay. In light of this, we examine X-ray micro-computed tomography (µCT) as an emerging complementary tool for the analysis of vascular calcification in animal models. We demonstrate that this non-destructive technique allows us to simultaneously quantify and localize calcification in an intact vessel in 3-D, and we consider recent advances in µCT sample preparation techniques. This review also discusses the potential to combine 3-D µCT analyses with subsequent 2-D histological, immunohistochemical, and proteomic approaches in correlative microscopy workflows to obtain rich, multifaceted information on calcification volume, calcification load, and signaling mechanisms from within the same arterial segment. In conclusion we briefly discuss the potential use of µCT to visualize and measure vascular calcification in vivo in real-time.

## 1. Introduction

Vascular calcification is an active, cell-regulated process leading to the formation of mineralized tissue, bone and/or cartilage within the blood vessel wall [1]. Most patients with cardiovascular disease have some calcification, although it is most prevalent in patients with chronic kidney disease (CKD), diabetes, and atherosclerosis. Vascular calcification is also associated with aging, and around 60% of 60-year old’s will have some degree of calcification [2]. Calcification is not only highly prevalent, but there is now substantial evidence that it contributes to the morbidity and mortality associated with these common conditions [3,4].

The arterial wall is composed of three layers: the tunica intima, tunica media, and tunica adventitia. Arterial intimal calcification occurs within atherosclerotic plaques, secondary to inflammatory mediators, and elevated lipids [5]. Intimal calcification can increase the risk of plaque rupture and subsequent cardiovascular events, although this is dependent on the size and localization of calcifications within the plaque. Microcalcifications (less than 50 µm in diameter) are postulated to appear first in atheromatous plaques, and then subsequently agglomerate to form macrocalcifications (more than 50 µm in diameter) [4,6]. While microcalcifications are believed to promote local stress and increase the risk of plaque rupture, extensively calcified plaques are considered to be less prone to rupture and adverse events [7]. The distribution of calcifications within an atheromatous plaque can also impact plaque stability. For example, spotty calcifications which are classified as several mid-sized (~0.5 mm in diameter) calcium deposits spaced 1–3 mm apart, are associated with ‘high-risk’ plaques in coronary and carotid arteries [8,9,10].

Arterial medial calcification occurs along the elastic lamellae of the tunica media and is associated primarily with aging, CKD, and diabetes [5]. Medial calcification reduces arterial (including aortic) elasticity, leading to an increase in pulse wave velocity, development of left ventricular hypertrophy, reduced coronary perfusion, and myocardial infarction and failure [11,12]. While intimal and medial calcification can occur independently of each other, both intimal and medial calcification are observed frequently in the same arterial segment in patients with CKD [13]. As these two types of arterial calcification can be observed simultaneously in the same blood vessel, their pathogeneses are believed to involve several common molecular and cellular mechanisms including the osteo/chondrogenic differentiation of vascular smooth muscle cells (VSMCs), VSMC apoptosis, loss of calcification inhibitors, calcifying matrix vesicle/exosome release, and matrix mineralization. However, other mechanisms specific to each type of calcification also appear to be involved [5]. For an in-depth review of the mechanisms that regulate the development of vascular calcification, readers are referred to other excellent articles [3,4,5,6].

This article aims to briefly review the different rodent models used to study arterial medial and/or intimal calcification in vivo and to discuss the strengths and weaknesses of the current techniques used to quantify calcification in these models, including the *o*-cresolphthalein assay and histology. This article will then explore the exciting opportunities presented by X-ray micro-computed tomography (µCT) to analyze and quantify vascular calcification in pre-clinical animal models. Recent advances in sample preparation techniques will be discussed, as will the potential for combining 3-D and 2-D work-flows in pre-clinical animal models to provide complementary information about calcification volume, calcification load, and signaling mechanisms from within the same arterial segment. Finally, this article will consider the use of µCT devices to visualize and measure vascular calcification in vivo, which could enable vascular calcification to be analyzed in real-time.

## 2. Rodent Models of Arterial Medial and Intimal Calcification

Rodent models of CKD are used widely to study arterial medial calcification and include interventions such as vitamin D_3_ overload, adenine administration, and 5/6 nephrectomy [14,15,16]. The calcification(s) observed in these models is often distributed focally and is ‘patchy’ in nature [17,18], which is similar to the patterns of calcification observed in specimens from patients with CKD [19,20,21]. Atherosclerotic apolipoprotein E knock-out (ApoE^−/−^) and low-density lipoprotein receptor knock-out (LDLR^−/−^) mice are popular models to study the development of arterial intimal calcification [22], with both micro- and macro-calcifications frequently detected in severe atheromatous plaques [23,24], as is the case in humans. CKD can also be induced in atherosclerotic ApoE^−/−^ or LDLR^−/−^ mice, leading to the formation of both arterial medial and intimal calcification in the same arterial segment [23,24,25]. These different rodent models of vascular calcification are summarized in Table 1. For an in-depth review of other research models for studying vascular calcification, readers are referred to Reference [16].

## 3. Commonly Used Methods to Quantify Vascular Calcification in Animal Models

To date, biochemical assays and/or histology have been used widely to quantify and visualize vascular calcification in pre-clinical animal models [17,18,26,27,28,29,34,41,46]. For the commonly-used *o*-cresolphthalein complexone method, the total tissue calcium content is eluted in acid, and the absorbance of the supernatant is measured at 570 nm using a plate reader [26]. However, acidic digestion destroys the tissue, and it is unknown whether increased calcium content is representative of actual calcification or just calcium excess. An additional limitation of the *o*-cresolphthalein assay is that it is unable to identify the localization and/or distribution of calcification throughout the arterial wall. This is of particular importance in intimal calcification studies as there is a clinical need to distinguish between macro- and micro-calcifications. Thus, biochemical quantification alone can be considered insufficient to evaluate the extent and potential consequences of vascular calcification.

**Figure 1:**
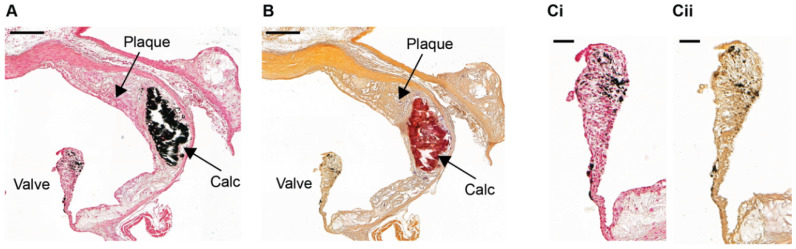
Detection of arterial intimal calcification in aortic root sections from an ApoE^−/−^ mouse. Serial sections of a formalin-fixed aortic root from a 10-week old ApoE^−/−^ mouse fed a high fat, high cholesterol diet for 28 weeks stained with (**A**), (**C**)/(**i**) von Kossa or (**B**), (**C**)/(**ii**) alizarin red S to detect calcification (Calc). Von Kossa-stained tissue sections were counterstained with nuclear fast red. False-positive black staining is detected in both the (**C**)/(**i**) von Kossa and (**C**)/(**ii**) alizarin red S stained aortic valve due to the presence of melanocytes. (**A**), (**B**) Scale bar = 200 µm. (**C**)/(**i-ii**) Scale bar = 50 µm.

Conventional 2-D histology combined with light microscopy allows specific tissue and cellular components to be identified and is, therefore, used to classify a wide range of tissue conditions and disease states. Formalin-fixed, paraffin-embedded tissues are routinely prepared for 2-D histopathologic investigations; with the von Kossa or alizarin red S stains being used to detect calcifications in these specimens [17,26,27,28,29,41,46,63]. Calcified tissue stains black for phosphate with von Kossa (Figure 1A) [64], and red for calcium with alizarin red S (Figure 1B) [65]. However, in mouse aortic valves, von Kossa can give a false positive stain due to the presence of melanocytes [6] (Figure 1(C)/(i)) which can also appear black in sections stained with alizarin red S (Figure 1(C)/(ii)) [66]. Therefore, it is advisable that both von Kossa and alizarin red S stains are used to confirm calcification in the aortic valves [67,68]. Alternatively, hematoxylin and eosin (H&E) staining could be used to identify melanocytes.

**Figure 2:**
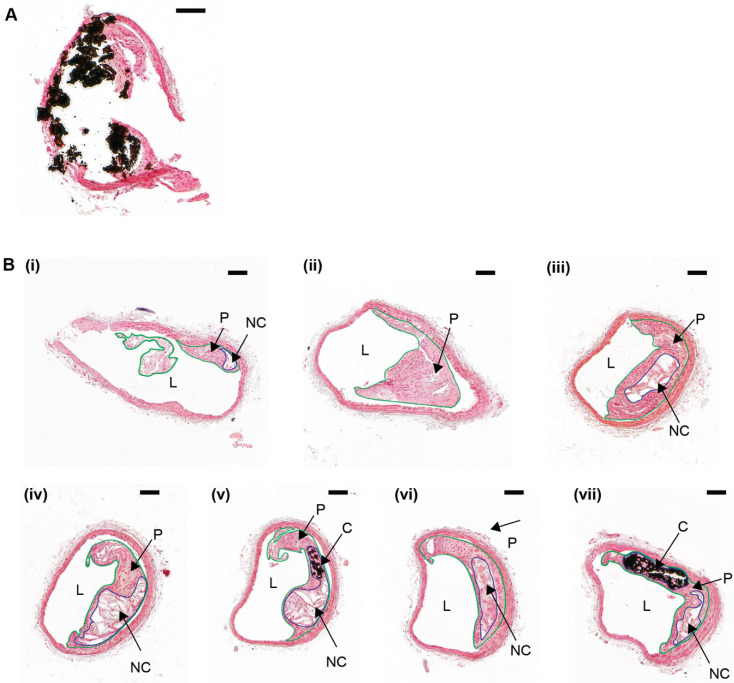
Atherosclerotic plaque calcification heterogeneity in a serially-sectioned brachiocephalic artery from an ApoE^−/−^ mouse. (**A**) Formalin-fixed brachiocephalic artery tissue section from a 10-week old ApoE^−/−^ mouse fed a high fat, high cholesterol diet for 28 weeks. The tissue was taken through a series of graded alcohols and rehydrated, stained with von Kossa, and then counterstained with nuclear fast red. A large calcification (stained black) has “dropped out” during sectioning, resulting in shearing artefacts and tissue damage. (**B**) A formalin-fixed brachiocephalic artery from a 10-week old ApoE^−/−^ mouse fed a high fat, high cholesterol diet for 28 weeks was serially sectioned, collecting a total of 120 sections, with each section—10 µm in size. Tissue sections were collected from the brachiocephalic artery bifurcation (**B**)/(**i**) into the main brachiocephalic artery trunk (**B**)/(**ii**–**vii**), and sections every 200 µm intervals were stained with von Kossa and counterstained with nuclear fast red. The atherosclerotic plaque is outlined in green. The necrotic core, which is an internal lipid-rich region lacking in collagen or cell nuclei but contains cell debris, is outlined in blue. Both atherosclerotic plaque and calcification heterogeneity are noted throughout the brachiocephalic artery. Lumen (‘L’); Plaque (‘P’); Necrotic core (‘NC’); Calcification (‘C’). Scale bar = 100 µm.

While the alizarin red S and von Kossa stains have been used successfully as effective clinical and research tools to detect micro- and macro-calcifications in blood vessels, these histological approaches rely on extended tissue preparation procedures; namely fixation, embedding, sectioning, and staining, which can modify the geometry of the vessel with respect to the in vivo situation. Formalin-induced amine cross-links and paraffin wax act in concert to chemically strengthen and physically support the tissue [69], yet the sectioning and staining processes invariably disrupt the structure of organs such as large arteries. In this regard, conventional histological sectioning can induce tears and folds in arterial tissue, and in the specific case of large arteries, often causes separation of the collagen-rich adventitia from the external elastic lamina [70]. In heavily calcified blood vessels such as those found next to extensive lipid-rich atherosclerotic plaques, calcified deposits are also prone to “drop out” of the arterial wall when sectioned, producing shearing artefacts and tissue damage (Figure 2A) [71,72]. This precludes the accurate quantification of calcification using image analysis tools such as Image J. To overcome these limitations, atherosclerotic blood vessels typically undergo decalcification processes before histological analysis, but this can cause tissue shrinkage and modify vessel morphology, and precludes the subsequent detection of calcified deposits in the blood vessel.

Several studies have also identified a marked variation in calcium deposition over relatively short lengths of a blood vessel [73,74]. This issue is amplified in calcified atherosclerotic lesions, in which diffuse microcalcifications and focal macrocalcifications can be identified within the same lesion (Figure 2(B)/(i–vii)) [75,76]. Therefore, it is necessary (but not always implemented) for multiple serial tissue sections to be cut, stained, imaged, and analyzed from a single blood vessel—this is laborious and time-consuming. As a result, experimental bias and error can occur by selecting areas of analysis either randomly along the profile of the vessel, or at the site of maximum calcification. Thus, key features such as micro-calcifications and extracellular matrix remodeling (e.g., elastin degradation [28,77,78]) may not always be detected.

Biochemical and histological assays have traditionally been used in combination to quantify and visualize vascular calcification in animal models [17,26,27,28,29,41,46]. However, these two approaches cannot be carried out on the same blood vessel, preventing complementary analyses of calcification volume and signaling mechanisms from being obtained from the same arterial segment. In light of this, alternative 3-D approaches that can be combined with 2-D histological measurements are required.

## 4. X-ray Micro-Computed Tomography (µCT)

In the clinic, vascular calcification imaging has mostly relied on medical computed tomography (CT), which achieves resolutions in the mm range [79]. While coronary artery calcium scores derived from these scans are predictive of atherosclerotic burden [80,81], the low resolving power of medical CT devices have made them unsuitable for pre-clinical applications in animal models of vascular calcification. Initially developed in the early 1980s, µCT devices can achieve resolutions in the sub-micron to 100 µm range and are finding increasing application in materials science [82,83,84,85], parasitology [86], and biomedical research [83,87,88,89,90].

In vivo CT imaging is usually carried out using instruments with a gantry-based scanning arrangement, where the X-ray source and detector are rotated around a stationary object [86]. This allows for simpler stabilization of the live, often anesthetized, specimen inside the instrument. However, the fixed source-sample arrangement limits the adjustments possible to improve image resolution. In contrast, the rotating table µCT is commonly used to image ex vivo tissues [86]. Here, the sample rotates while the X-ray source and detector remain stationary. Both synchrotron [91,92,93] and laboratory [70,73,74,92,94,95,96] X-ray sources can be used to image ex vivo tissue, including blood vessels [70,73,91,94,97]. Synchrotron instruments normally use an essentially parallel beam of X-rays, whereas a cone beam is more typical of laboratory instruments. In the latter case, the X-ray source to sample and sample to detector distances can be varied, allowing the researcher to adjust the magnification and field of view to the sample size, and thus, the optimize image resolution. The coherence of synchrotron sources means that they typically offer phase contrast, in addition to attenuation contrast, which can be useful for obtaining sufficient contrast in soft tissue samples such as blood vessels [87,97]. However, only a limited number of synchrotron facilities exist in the world, which is associated with high running costs and access constraints. In contrast, laboratory µCT instruments tend to provide lower flux (less chance of dose-induced damage) and are more easily accessed.

During CT scanning, around 500–4000 2-D radiographs (projections) are acquired of the sample taken from a series of viewing angles over 180 °C or 360 °C. Then, CT reconstruction algorithms are used to reconstruct a grayscale 3-D volume, in which materials of different X-ray attenuation are represented as different voxel intensities [86,98]. Visualization and image processing software can then be used to analyze the reconstructed data further. Given sufficient differences in X-ray attenuation of the constituent materials, for example, between calcifications and soft tissue, structures can be visualized immediately with little manual input, thereby removing any potential experimental bias.

The 3-D image volume can then be virtually sliced in any direction and interrogated similarly to the physical serial sectioning of a tissue block, with a slice spacing equal to the CT voxel size. Using 3-D data visualization and analysis software from open (e.g., Image J (U.S. National Institute of Health, MD, USA); Drishti (National Computational Infrastructure VizLab, Canberra, Australia)) or proprietary sources (e.g., Avizo (Thermo-Fisher Scientific, Waltham, MA, USA); VGStudio (Volume Graphics, Heidelberg, Germany); Dragonfly (Open Research Systems, Montreal, Canada); Simpleware (Synopsys, Mountain View, CA, USA)), image stacks and orthogonal plane renderings (xy, yx, and xz) of µCT data can be used to provide a detailed overview of tissue microarchitecture, as well as to view and quantify the orientation, heterogeneity, and connectivity of microscopic tissue structures in 3-D. The histology equivalent of these analyses would require serial sectioning of the tissue block in its entirety, which is time consuming and prone to sectioning artefacts. For example, 500 histological sections would be required to build up a 3-D picture of a fixed 3 mm long brachiocephalic mouse artery based on 6 µm thick sections. In contrast, a comparable volume could be acquired non-destructively in less than 7 min for high resolution synchrotron-based µCT instruments (1.1 µm voxel size [91]) and less than 1 h for laboratory µCT instruments (7–10 µm voxel size) [73,94], without any user intervention or sample preservation issues.

Some of the limitations of µCT imaging include the need for samples to be fixed and physically stable (i.e., not moving) for the duration of the scan (see Section 6—Micro-CT sample preparation techniques). The field of view is also related to spatial resolution, which means that high-resolution scans only image a limited volume of material. While samples larger than the field of view are permissible in µCT scanning (e.g., during region-of-interest scanning), the sample size within a µCT instrument is nonetheless restricted. Further challenges, such as image contrast and image artifacts, are discussed below (see Section 5).

## 5. Enhancing Contrast in Soft Tissue µCT

Mineralized tissues such as bone attenuate a large fraction of the incident X-ray photons producing good image contrast. In contrast, soft tissues such as blood vessels have inherently low X-ray attenuation contrast between the tissue and the supporting material used for sample immobilization. Heavy metal stains such as osmium tetroxide [99], phosphotungstic acid [70,100,101,102], and iodine [70,101,102,103] have been used to overcome this potential limitation. These stains differentially bind to internal regions of a sample, providing increased contrast due to the higher range of X-ray attenuation [87,101]. However, staining duration is often sample- and stain-specific, and it is therefore, essential that staining concentration and duration is optimized for a particular tissue type [104].

Osmium tetroxide preferentially binds to lipids and has been used to visualize coronary arteries in mouse hearts [99]. However, its high toxicity makes it impractical for routine use. While iodine-potassium iodide and phosphotungstic acid can be perfused through a whole mouse within 30 min to visualize the vasculature in situ by µCT, these stains show a weak affinity for the arterial wall and can diffuse into the surrounding tissue [101,102]. Therefore, a novel µCT stain that preferentially binds to the arterial wall and provides good µCT contrast is required. Dunmore-Buyze et al. [101] recently generated a modified Verhoeff’s stain (iodine, aluminum, and iron) and demonstrated that it binds preferentially to the arterial wall of medium and large arteries of intact mice; enabling the visualization of the whole vasculature in situ by µCT for the first time.

Selected X-ray contrast agents such as phosphotungstic acid are incompatible with subsequent histological staining protocols, including H&E and Weigert’s elastic fiber stain due to blockage of the metal-binding sites utilized by these stains [70]. An overview of the different X-ray contrast agents that have been used in pre-clinical vascular studies are summarized in Table 2. 

Soft tissue µCT protocols that do not utilize these X-ray contrast agents are, therefore, preferable in a correlative 3-D and 2-D imaging workflow. Phase-contrast µCT has been used to image unstained blood vessels [70]. It exploits a shift in the phase of the X-ray beam when the tissue being observed causes slight changes in the refractive index [105]. Experimentally, phase-contrast relies on coherent beams such as those generated at synchrotron facilities, or the use of special optical elements such as gratings [106]. However, some laboratory systems have sufficient beam coherence to generate phase-contrast, and thus, significantly enhance the contrast of soft tissue [70,107]. 

Micro-CT imaging methods can be affected by artefacts in the reconstructed images. In particular, streak artefacts arise when imaging materials of very different X-ray attenuation, such as soft tissue and calcifications [95]. This artefact occurs when the dense calcified regions attenuate the X-ray beam strongly, masking the illumination of lower attenuating soft tissue. This is especially problematic when the boundary between the two materials is itself of interest, for example, in calcified blood vessels [95]. While the artefact itself can be reduced by utilizing higher beam energy, this can also reduce contrast in the lower attenuating material, as the energy-dependent X-ray attenuation coefficients become more similar at higher beam energies. Methods for improving image quality in the cases where the constituent materials have very different attenuation properties remains an area of active research [111]. Most laboratory instruments utilize a polychromatic beam, leading to beam hardening artefacts due to the due to the progressive hardening (shift of the energy spectrum to higher energy) of the beam as the low energy X-rays become attenuated on passing through attenuating phases. However, due to the low X-ray attenuation and non-uniformity of typical biological tissue samples, beam hardening artefacts are rarely of much practical relevance.

## 6. Micro-CT Sample Preparation Techniques

During µCT image acquisition, tissue sample movement during sample rotation has the potential to cause the corresponding 2-D projections to be out of the registry, thereby producing poorly reconstructed 3-D volumes. Several protocols for sample immobilization have been developed to overcome this problem for small soft tissues such as blood vessels including: (i) liquid immersion protocols using alcohol [89,100,112] or corn oil [73,94]; as well as tissue embedding protocols using (ii) agarose gels [90,113,114,115], (iii) plastic embedding resins [116], and (iv) paraffin wax [70,76,88,91,95,96,117]. The advantages and disadvantages of these different sample immobilization techniques are summarized in Table 3.

### 6.1. Liquid Immersion

Sample immobilization in liquids has been utilized for several soft tissues during µCT imaging, including blood vessels [73,89,94,100,112]. Huesa et al. [73,94] reported that µCT imaging could be utilized to visualize and quantify arterial medial calcification in formalin-fixed aortae from Ecto-nucleotide pyrophosphatase/phosphodiesterase-1 knock-out (Enpp1^−/−^) mice; a model of generalized arterial calcification of infancy. Mouse aortae were immersed in an iopamidol-based contrast agent (Niopam300; Brako UK Ltd., Buckinghamshire, UK), and subsequently immersed in corn oil for scanning. The corn oil offered a different density to water providing a clearer definition of the soft arterial wall tissue and enhanced quantification of the blood vessel wall and calcification volumes. However, these liquid immersion protocols are impractical for long-term sample storage and can be associated with sample movement during imaging. Subsequent histological and immunohistochemical analysis of the tissue may also be hampered.

### 6.2. Agarose Gels

Agarose gel preparation techniques offer improved tissue sample immobilization when compared to liquid immersion protocols, and 1% (*w*/*v*) agarose gels have been successfully used for ex vivo blood vessel immobilization during µCT image acquisition [115]. However, agarose gels are unsuitable for long-term storage, and the difficulties associated with recovering intact tissue samples from agarose gels can often hamper subsequent histological 2-D analyses.

### 6.3. Plastic Resins

Plastic embedding resins have been explored more recently, with LR White acrylic resin (Sigma-Aldrich, UK) offering a low tendency to form bubbles during polymerization, long-term preservation of millimeter-scale specimens, and rigid immobilization for µCT [116]. A drawback to this technique is that the squared edges of plastic resin blocks are frequently associated with edge diffraction artefacts that can interfere with imaging. Lin et al. [116] demonstrated that X-ray transparent polyimide tubing could be used as a tissue mold to create rounded samples without edges to overcome this. The spatial resolution is also improved by restricting the volume of resin around the tissue sample. As the voxel size is directly related to the field of view, smaller samples generally allow for higher resolution. Region of interest scans within larger samples are often possible. However, sample material outside the field of view will contribute to noise and reduce absorption contrast within the scanned region, which again makes small sample sizes advantageous.

### 6.4. Paraffin Wax

Formalin-fixed, paraffin-wax embedded blood vessels have become increasingly popular in ex vivo µCT applications [70,76,88,91,95,96,117]. Specifically, rodent blood vessels can be formalin-fixed and paraffin wax-embedded for both laboratory and synchrotron-based µCT sources without the need for X-ray contrast agents, achieving isotropic voxel sizes between 0.7 and 20 µm, depending on sample size and field of view [70,91,95,96]. The similar X-ray absorption coefficient of paraffin wax and biological tissue [118] can lead to small differences in the absorption contrast between the two materials, especially when a large amount of wax surrounds a small sample. However, this limitation can be overcome by manually trimming paraffin wax-embedded tissue blocks [91]. Alternatively, tygon tubing can be used as a mold to minimize the thickness of the external paraffin wax and to create samples with rounded edges [70]. The small paraffin wax-embedded samples can then be re-embedded into larger blocks to allow for subsequent histological sectioning following non-destructive µCT scanning.

Formalin-fixed, paraffin-wax sample preparation techniques offer several advantages. The use of chemical fixatives, combined with mechanical paraffin support, can provide a stable medium for the collection of high-quality 3-D µCT data without movement artefacts. Tissue samples can be prepared using standard histological procedures, enabling their compatibility with subsequent 2-D histological and immunohistochemical analyses [70,95]. Laser capture microdissection coupled to mass spectrometry (LCM-MS) can also be performed using formalin-fixed, paraffin wax-embedded tissue sections. Indeed, this method is currently being optimized for microproteomics to identify and quantify proteins in specific regions of tissue [119,120,121]. Compared with other µCT sample preparation techniques, high-quality 3-D µCT data can, therefore, be collected from formalin-fixed, paraffin-wax embedded tissue samples to identify volumes of interest for subsequent (and also compatible) histological, immunohistochemical, and proteomic analysis within a correlative workflow.

## 7. Combining 3-D and 2-D Vascular Calcification Workflows in Pre-Clinical Models

**Figure 3:**
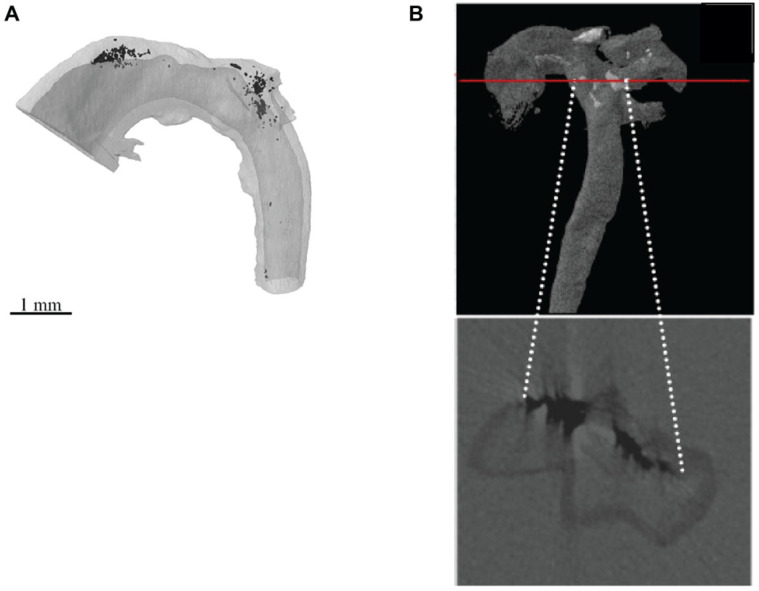
µCT imaging of calcified mouse blood vessels. (**A**) A formalin-fixed, corn oil immersed aortae from the Enpp1^−/−^ mouse model of arterial medial calcification was scanned by µCT using a voxel size of 10 µm, and the images reconstructed to form a 3-D imaged volume. X-ray dense calcifications (black) localized to the upper curvature of the ascending aorta and aortic arch. (**B**) A formalin-fixed, paraffin-wax embedded aorta from the LDLR^−/−^ mouse model of atherosclerosis and arterial intimal calcification was scanned by µCT using a voxel size of 5.63 µm, and the images reconstructed to form a 3-D imaged volume. X-ray dense calcifications (black) are observed in a phase virtual slice from the 3-D imaged volume (indicated by the red line). (**A**) Reproduced from Reference [73] with permission. (**B**) Reproduced from Reference [95] with permission.

To date, only a handful of studies have utilized laboratory µCT sources to analyze vascular calcification in animal models [73,94,95,96,122]. Arterial medial calcification has been quantified using µCT in formalin-fixed, corn oil immersed aortae from Enpp1^−/−^ mice, achieving an isotropic voxel size of 7–10 µm (Figure 3A) [73,94]. However, a limitation of these studies was the inability to correlate the 3-D µCT data with subsequent 2-D histological analyses. To overcome this, Awan et al. [95,96] used paraffin-wax embedded aortas from atherosclerotic LDLR^−/−^ mice to combine 3-D volumetric calcification analyses with 2-D histological analyses in the same arterial segment (Figure 3B). However, the low-resolution capabilities achieved (isotropic voxel size between 5 and 20 µm) in these studies prevented the visualization of the finer structures of the calcifications and the blood vessel wall extracellular matrix (ECM).

**Figure 4:**
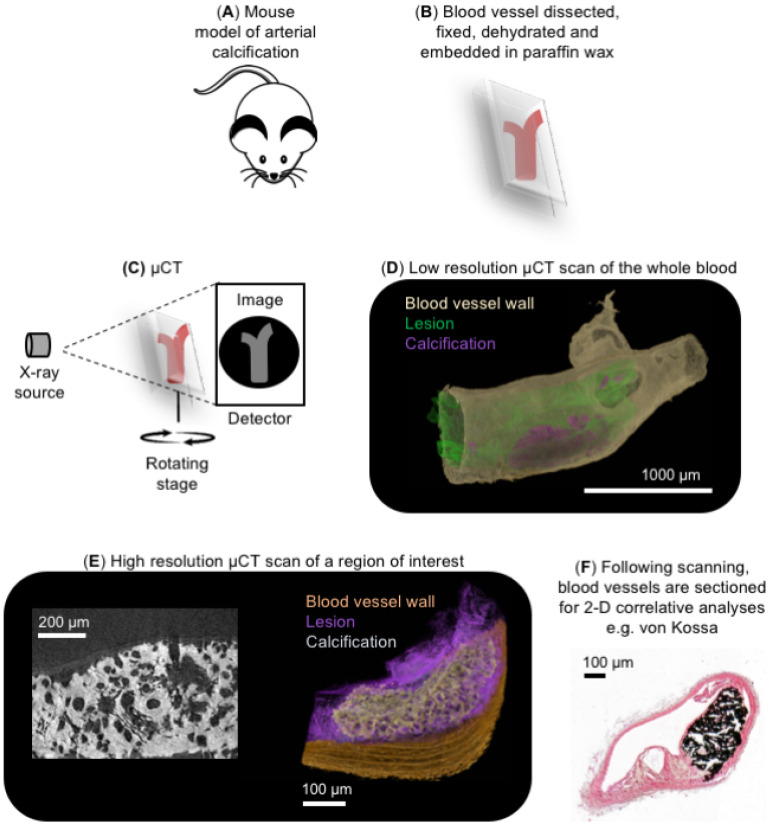
Correlative imaging of ex vivo blood vessels. (**A**,**B**) Blood vessels dissected from mouse models of arterial calcification are fixed in 10% (*v*/*v*) neutral buffered formalin at 4 °C overnight. Tissues are dehydrated and paraffin wax-embedded using standard histological techniques. Paraffin blocks are then manually trimmed of embedding surplus to improve spatial resolution and reduce edge diffraction artefacts [91]. (**C**) Blood vessels are imaged on a laboratory µCT system; in this example, a Carl Zeiss XRM Versa-510 was used. A schematic illustration of the main components of the µCT system is shown. (**D**) A low-resolution µCT scan of the complete artery cross-section is performed to generate a 3-D reconstruction (achieving voxel sizes between 3 and 3.8 µm), followed by (**E**) a higher resolution region-of-interest scan to visualize the finer structures of the calcified deposits and vascular extracellular matrix (ECM) (achieving voxel sizes between 0.50 and 0.74 µm). X-ray dense calcification is shown in white in the phase virtual slice. In this example, images have been collected from a brachiocephalic artery taken from a 10-week old ApoE^−/−^PKCα^−/−^ mouse fed a high fat, high cholesterol diet for 28 weeks to induce atherosclerosis and intimal calcification. Markers such as gold spheres could be used to locate the same region of interest across µCT and nCT devices to analyze tissue structures across multiple length scales [70,87]. (**F**) Following µCT scanning, blood vessels can be re-embedded in paraffin wax and sectioned for histological (e.g., von Kossa to analyze calcification), immunohistochemical and LCM-MS approaches.

To overcome the poor resolution capabilities for vascular calcification studies, previously described multi-scale scanning µCT approaches [70,87] have been used to visualize and quantify calcified deposits in blood vessels to achieve isotropic voxel sizes down to 0.5 µm (Figure 4A–E). To do this, dissected blood vessels that have been formalin-fixed, dehydrated, and embedded in paraffin wax using standard histological approaches (Figure 4A,B) [70,91] are scanned using a laboratory µCT system (Figure 4C) [95,96]. A faster, lower resolution µCT scan of the whole blood vessel is performed initially to visualize the localization and distribution of calcified deposits in 3-D (Figure 4D), which can then be quantified [95,96]. Regions of interest are then subsequently imaged at higher resolutions to examine the finer structure of the calcified deposits and the blood vessel ECM (Figure 4E) [70]. By taking advantage of higher objectives, these higher resolution scans can be performed using laboratory µCT sources to achieve a spatial resolution down to 0.5 µm (Figure 4E) [70]. Alternatively, sub-micron (e.g., 150 nm) resolutions could be achieved with laboratory nano-CT (nCT) sources, which has previously enabled the visualization of the medial interlamellar regions in rat carotid arteries [70]. Paraffin wax-embedded blood vessels can be subsequently mechanically sectioned for correlative histological, immunohistochemical, and laser capture microdissection techniques for nucleic acid or proteomic analyses (Figure 4F) [70,95,96].

As discussed in the Introduction, the structure and composition of an atheromatous plaque and, specifically, the size and distribution of intimal calcifications within the plaque are important risk factors for rupture and subsequent cardiovascular events [7,8,9,10,123]. Using 2-D histological approaches to analyze intimal calcification in pre-clinical animal studies has been associated with several drawbacks, including shearing artefacts and the inability to examine spatial heterogeneity in calcification throughout an intact blood vessel. Non-destructive µCT can overcome these limitations, enabling the 3-D visualization and quantification of lipids, calcification, adipose tissue, and the blood vessel wall in chemically-fixed, paraffin wax-embedded atherosclerotic human carotid [124,125], and coronary arteries [74,76,88,92,126] ex vivo. Importantly, µCT has also enabled the detection of microcalcifications in intact human blood vessels, which are associated with an increased risk of plaque rupture [74]. These microcalcifications are likely to go undetected using 2-D histological approaches unless serial tissue sections are cut, stained, imaged, and analyzed from a single blood vessel.

**Figure 5:**
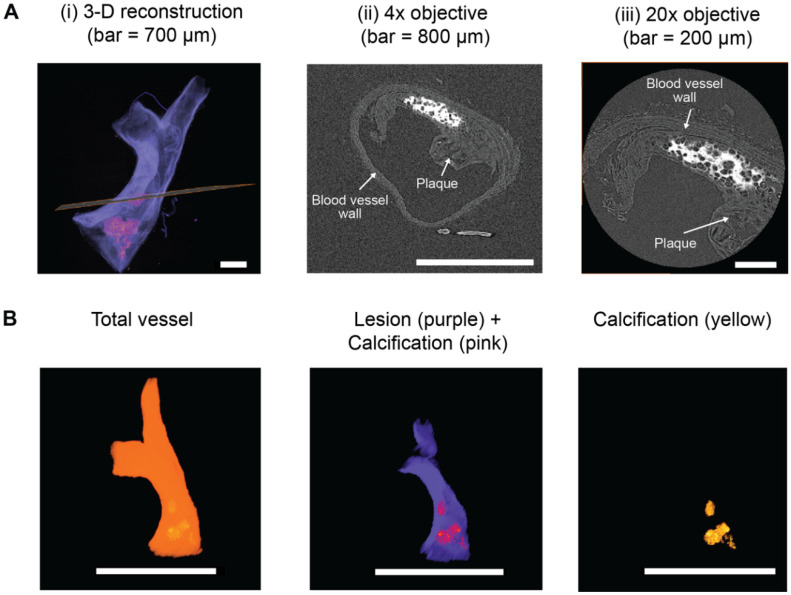
3-D µCT segmentation of calcified blood vessels. A brachiocephalic artery from a 10-week old ApoE^−/−^ mouse fed a high fat, high cholesterol diet for 28 weeks was scanned by µCT using a voxel size of 3 µm, and the images were reconstructed to form a 3-D imaged volume (**A**)/(**i**). A phase virtual slice from the 3-D imaged volume (indicated by the orange line) is shown in part (**A**)/(**ii**). X-ray dense calcifications (white) are evident within the plaque. (**A**)/(**iii**) A phase virtual slice from the same region scanned by µCT using a voxel size of 0.5 µm. (**B**) As there is low absorption contrast between the blood vessel wall and plaques, thresholding methods cannot be applied to segment these two volumes. Therefore, scans were manually segmented into the total blood vessels and plaque volumes. Thresholding was applied to segment the X-ray dense calcifications from the plaque. Scale bar = 2000 µm.

Thresholding methods have been applied to segment and quantify X-ray dense calcifications in the blood vessel wall and/or atheromatous plaques [74,122]. These are in good agreement with estimates from 2-D histological analyses [95,122]. However, the X-ray attenuation of the blood vessel wall and plaque is similar, resulting in a low absorption contrast between the two (Figure 5(A)/(ii,iii)) [76,97]. As a result, the plaque and blood vessel wall volumes currently have to be manually segmented in atherosclerotic blood vessels for quantification (Figure 5B) [97,100]. Going forward, the ability to interrogate 3-D X-ray data sets of calcified blood vessels into manageable and timely analysis platforms will enable µCT to become a routine partner in the analysis of vascular calcification in animal models of this pathology.

**Figure 6:**
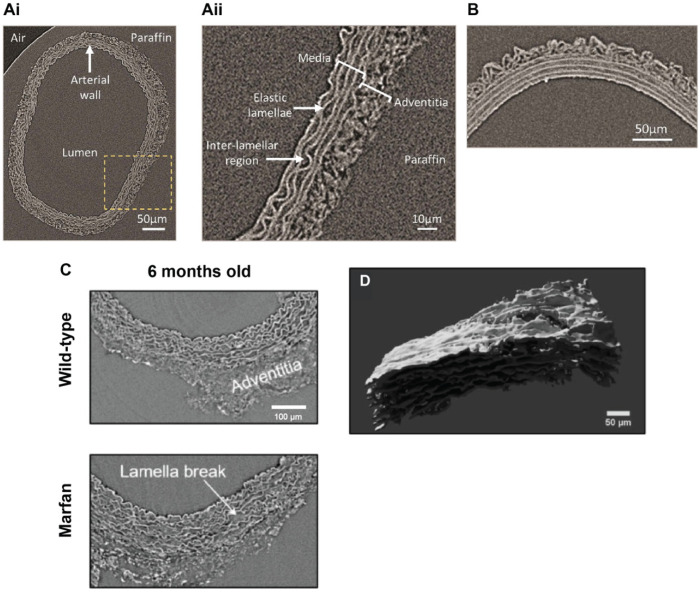
Visualization of elastic fiber remodeling in blood vessels. (**A**)/(**i**) Phase virtual slices from laboratory-based µCT of an intact rat common carotid artery at a 4x objective (voxel size 0.75 µm). The yellow box indicates the magnified region shown in (**A**)/(**ii**). (**B**) The resolutions achieved with laboratory-based µCT can be improved using a 20× objective (voxel size 0.5 µm). (**C**) Representative phase virtual slices from synchrotron-based µCT of intact aortas from 6 months old wild-type and Marfan syndrome mice. (**D**) 3-D volumetric rendering of a laterally-viewed aortic wall from a Marfan syndrome mouse; here, only lamellae of the tunica media layer are rendered. (**A**)/(**i**,**ii**), (**B**) Images reproduced from Reference [70]. (**C**,**D**) Images reproduced from Reference [91].

Elastin degradation has been associated with medial calcification in both humans and pre-clinical animal models and it has traditionally been analyzed by 2-D histology [28,77,78]. However, these analyses are limited by the artefacts that can be induced by sample sectioning or staining. Laboratory µCT devices are capable of visualizing the elastic lamina in healthy rat carotid arteries, achieving voxel sizes up to 0.75 µm with a 4× objective (Figure 6(A)/(i,ii)) and to 0.5 µm with a 20× objective (Figure 6B) [70]. Phase-contrast synchrotron-based µCT imaging has also been employed to visualize vascular remodeling in Marfan syndrome mice (Fibrillin-1^C1039G/+^), using a bespoke image processing package to segment and then quantify age- and disease-related changes in vessel diameter, medial layer thickness, and elastic lamellae spacing [91] (Figure 6C,D). Micro-CT could offer the opportunity to study ECM changes in calcified blood vessels in both 2-D and 3-D, thereby providing new mechanistic insights into vascular calcification.

Streak artefacts can be detected in heavily calcified blood vessels imaged by µCT [95] and can subsequently interfere with image analysis. While these streak artefacts can be circumvented by decalcifying the blood vessel before µCT imaging, this process can cause damage to the blood vessel wall, and the remaining decalcified plaque can infiltrate the vessel lumen [92]. Decalcification of diseased blood vessels is therefore not advised for µCT, particularly as calcifications cannot be visualized or quantified.

The correlative imaging workflow depicted in Figure 4 offers several advantages over the commonly-used 2-D histological and biochemical assays for the analysis of vascular calcification in animal models of this pathology (Table 4). 3-D µCT can detect both arterial medial and arterial intimal calcification in structurally intact blood vessels ex vivo, and the calcifications can be quantified accurately using thresholding methods. The high spatial resolution capabilities that can now be achieved on laboratory µCT instruments also enables the detection of microcalcifications [74], which cannot be reliably visualized and quantified using histological and biochemical assays. Furthermore, combining µCT analyses with subsequent 2-D histological, immunohistochemical, and proteomic approaches provides a valuable opportunity to obtain complementary information on calcification volume, calcification load, and signaling mechanisms from within the same arterial segment. This imaging workflow could, therefore, markedly increase the level of information obtained from a single animal and lead to reductions in the total number of animals required for these studies, meeting the requirements of the 3Rs of animal research (replacement, reduction, and refinement).

## 8. Future Outlook: In Vivo Imaging in Preclinical Animal Models

Micro-CT is gaining attention as a novel imaging modality to monitor the development of vascular calcification in vivo [127,128,129,130]. In line with the 3Rs of animal research, in vivo µCT has the potential to assess changes in calcification in long-term intervention studies without the need to sacrifice animals at multiple time-points. However, the use of in vivo µCT in rodent vascular calcification studies to date has been limited by poor resolution capabilities in the range of 35–200 µm [127,128,129,130]. This has limited the detection of microcalcifications in vivo. Given the clinical consequences of micro-calcifications in atherosclerotic plaques [131], µCT scanners, which can detect and distinguish between these two types of calcifications in vivo are required.

**Figure 7:**
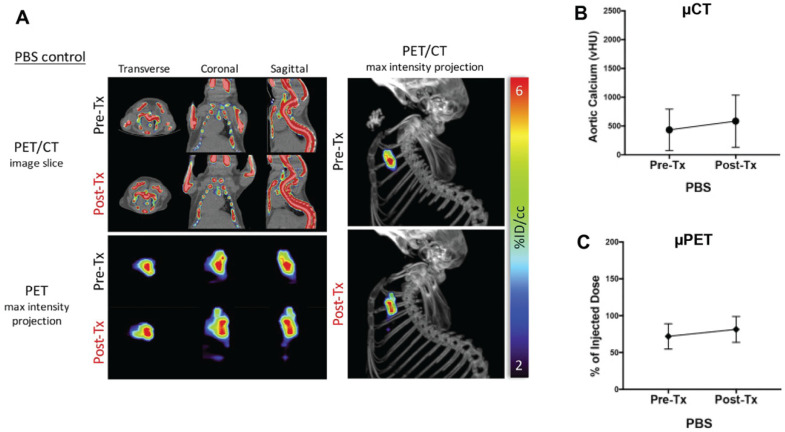
In vivo ^18^F-NaF µPET/CT Imaging. (**A**) Fused µPET/CT Imaging showing ^18^F-NaF uptake (representing calcium mineral surface area) in 12-month old phosphate-buffered saline (PBS)-treated mice, before and after treatment with teriparatide (Tx). Transverse, coronal, and sagittal slices of the chest are shown, with corresponding views of maximum-intensity projections of the mediastinal regions of interest. In the images on the right, a lateral view of the µPET maximum-intensity projection superimposed on the µCT image of the skeleton. (**B**) Quantification of aortic volumetric calcium deposition (vHU) measured before and after Tx treatment by μCT. (**C**) Quantification of aortic ^18^F-NaF uptake measured before and after Tx treatment by µPET. Reproduced from Reference [130] with permission.

^18^F-sodium fluoride micro-positron emission tomography (^18^F-NaF µPET) is a sensitive and specific method for detecting calcifications in blood vessels and offers a novel opportunity to discriminate between micro- and macro-calcifications in pre-clinical animal models in vivo [7,130,132]. ^18^F-sodium fluoride binds to the surface of hydroxyapatite crystals, where the hydroxyl ion is exchanged with the ^18^F ion to form fluorapatite. Importantly, ^18^F-NaF has been shown to bind calcifications in murine blood vessels that have not been readily detected by µCT [130,132]. These microcalcifications have a large surface area relative to the amount of calcium, and vascular ^18^F-NaF uptake is suggested to be a measure of the total calcification surface area and/or degree of metabolic activity [4,133]. In contrast, µCT is a measure of calcium mineral content regardless of surface area or metabolic activity. Therefore, combining µCT and ^18^F-sodium fluoride µPET could offer a novel opportunity to analyze both micro- and macro-calcifications in pre-clinical models of vascular calcification in vivo (Figure 7A–C) [130,132].

## 9. Conclusions

Historically, vascular calcification has been analyzed in pre-clinical animal studies using 2-D histological and biochemical assays. However, these techniques have several drawbacks, including sectioning-induced artefacts, and the inability to simultaneously visualize spatial heterogeneity and quantify calcification in a whole blood vessel. While non-destructive 3-D µCT has been used in a few pre-clinical vascular calcification studies to date, its increasing availability and recent advancements in µCT sample preparation techniques offer a novel opportunity to both visualize and quantify arterial medial and arterial intimal calcification in structurally intact blood vessels ex vivo. The high spatial resolution capabilities that can now be achieved on laboratory µCT instruments allow micro- and macro-calcifications to be distinguished and quantified. Moreover, vascular ECM changes such as elastin degradation can now be examined. Importantly, 3-D µCT analyses can also be combined with subsequent 2-D histological, immunohistochemical, and proteomic approaches, offering an exciting opportunity to obtain complementary information on calcification volume, calcification load, and signaling mechanisms from within the same arterial segment, from a single animal. Looking to the future, technological improvements in µCT scanners for in vivo studies could open up the exciting possibility to visualize and measure micro- and macro-calcifications in whole animals, revolutionizing the analysis of vascular calcification in real-time. Combining µCT with ^18^F-NaF/µPET within a correlative workflow could also enhance the detection of active microcalcifications in vivo and increase our understanding of how different signaling pathways and pharmacological agents regulate the development of calcification in pre-clinical animal models.

## Figures and Tables

**Table 1:** Rodent models of vascular calcification.

Model (Disease)	Calcification	Overview	Advantages	Limitations
5/6 nephrectomy & high phosphate diet (CKD)	Medial	A reduction in (functional) renal mass is performed, usually using a 2-stage surgical procedure: (i) a partial nephrectomy is performed by either electrocautery, resection or ligation of the upper and lower kidney poles, or ligation of 2/3 of the extrarenal artery; (ii) following 1-2 weeks recovery, a uninephrectomy is performed on the contralateral kidney [17,26,27,28,29,30,31]. Animals are then fed a chow diet supplemented with phosphate (0.9–1.5%) for up to 16 weeks to induce arterial medial calcification [27,28,29].	➢ Widely used in both mice and rats [17,26,27,28,29,30,31]. ➢ Animals develop arterial medial calcification in the absence of atherosclerosis and inflammation. ➢ High phosphate diets can be supplemented with vitamin D_3_ to accelerate the development of arterial medial calcification [17,29].	➢ A two-stage surgical procedure is required to reduce the risk of acute kidney injury. ➢ Mice on the DBA/2J mouse genetic background are recommended as they have a greater susceptibility to developing calcification [17,29,30]. ➢ Female DBA/2J mice are more susceptible to developing calcification compared with males [27,32,33].
Vitamin D_3_ overload (CKD)	Medial	Supra-physiological doses of vitamin D_3_ result in acute hypercalcemia and hyperphosphatemia, leading to arterial medial calcification. Vitamin D_3_ is administered via intraperitoneal or subcutaneous injections to induce calcification in C57BL/6 mice [34,35,36]. In rats, vitamin D_3_ is administered orally [37], subcutaneously [38], or intramuscularly [39].	➢ Does not require surgery. ➢ Widely used in both mice and rats [34,35,37,39,40]. ➢ Arterial medial calcification can be detected within 14 days [34,35].	➢ Vitamin D_3_ is commonly used in combination with nicotine [40] or the 5/6 nephrectomy model [37,39] to induce calcification in rats.
Adenine (CKD)	Medial	Administration of adenine to rats via 0.15–0.75% adenine-rich diets [41,42,43] or oral gavage [44] leads to the formation of crystals in renal tubules, resulting in tubular injury, obstruction, marked fibrosis and arterial medial calcification [45]; the development of calcification is accelerated by supplementing adenine-rich diets with calcitriol [43] and/or 1–1.2% phosphate [41,43]. Chow diets supplement with 1.2–2% phosphate and 0.25% adenine have also been reported to induce arterial medial calcification in both DBA/2 [46] and C57BL/6J mice [41].	➢ Does not require surgery. ➢ Widely used in both mice and rats [41,44,46,47]. ➢ Suitable for mice on different genetic backgrounds [41,46].	➢ Weight loss ensues in rats fed a 0.75% adenine-rich diet [42,48]. ➢ In rats fed a 0.75% adenine-rich diet, only 30% of animals are reported to develop calcification [42]; the extent and frequency of calcification can be improved by using 0.75% adenine-rich diets with a low protein content (2.5%) [42].
ApoE^−/−^ mice (Atherosclerosis)	Intimal	ApoE^−/−^ mice spontaneously develop elevated plasma VLDL and LDL levels, leading to the formation of atheromatous plaques and intimal calcification in the aortic root and ascending aorta as they age. The development of more wide-spread plaques requires high fat, high cholesterol feeding [49,50,51,52]. Male ApoE^−/−^ mice typically develop intimal calcification after 18–28 weeks of high fat diet-feeding [52,53,54].	➢ Atherosclerosis and calcification are accelerated by high fat, high cholesterol feeding [52,53]. ➢ ApoE^−/−^ mice develop more extensive atherosclerosis and calcification than LDLR^−/−^ mice [49].	➢ The C57BL/6J genetic background is recommended as these mice are more susceptible to developing atherosclerosis and intimal calcification [33,55]. ➢ There is a lack of data on female ApoE^−/−^ mice, as most studies have focused on males alone.
LDLR^−/−^ mice (Atherosclerosis)	Intimal	LDLR^−/−^ mice develop familial hypercholesterolemia, which is characterized by elevated LDL. Minimal plaques develop in the aorta of LDLR^−/−^ mice fed a chow diet, but large atherosclerotic plaques develop over the entire length of the aorta with high fat, high cholesterol feeding. LDLR^−/−^ mice develop intimal calcification after 15-30 weeks of high fat diet-feeding [56,57,58].	➢ LDLR^−/−^ mice typically show a modest doubling of plasma cholesterol levels and represent a moderate model of atherosclerosis and intimal calcification. ➢ Atherosclerosis and calcification development can be accelerated by high fat, high cholesterol feeding [58]. ➢ Both male and female mice have been used [56,57].	➢ The C57BL/6J genetic background is recommended as these mice are more susceptible to developing atherosclerosis and intimal calcification [33,55].
5/6 nephrectomy & ApoE^−/−^ or LDLR^−/−^ mice (CKD & atherosclerosis)	Medial & intimal	Uremia is induced in ApoE^−/−^ [23,25,59,60,61] or LDLR^−/−^ [24,62] mice by performing a 2-stage 5/6 nephrectomy. High fat, high cholesterol feeding has been used in 5/6 nephrectomy LDLR^−/−^ mouse studies to accelerate atheromatous plaque and calcification formation [24].	➢ Mice develop both arterial medial and intimal calcification in the same arterial segment, as observed in humans [13,23,25,60,61]. ➢ Calcification is detected in 5/6 nephrectomy ApoE^−/−^ mice without the need for a high fat, high cholesterol diet [23,25,60,61]. ➢ Male and female 5/6 nephrectomy LDLR^−/−^ mice have been reported to develop plaques at a similar rate [24].	➢ Two-stage surgical procedure. ➢ The C57BL/6J mouse genetic background is preferred [24,59,62]. ➢ Female ApoE^−/−^ mice are more susceptible to developing calcification compared with males in this model [23,60,61].

**Table 2:** Overview of some of the X-ray contrast agents which have been used in vascular studies.

Contrast Agent	Advantages	Limitations
Osmium tetroxide [99]	➢ It can be used to visualize coronary arteries in mouse hearts [99].	➢ Highly toxic. ➢ Tissue penetration is slow [108]. ➢ It does not work well on tissues that have been preserved in alcohol [108]. ➢ Expensive to purchase.
Phosphotungstic acid [70,100,101,102]	➢ Stable stain. ➢ It can provide visualization of the aorta in situ by µCT [101]. ➢ Compatible with immunofluorescence staining [70]. ➢ Has been used in both whole animal and ex vivo tissue application [70,100,101,102].	➢ Tissue penetration can be slow [70]. ➢ Weak affinity for the arterial wall [101]. ➢ It can cause some tissue shrinkage [109]. ➢ Not compatible with H&E and Weigert’s (elastin) staining protocols [70].
Iodine [70,101,102,103]	➢ Rapid tissue penetration. ➢ Low toxicity and inexpensive. ➢ It can provide visualization of the aorta and coronary arteries in situ by µCT [101]. ➢ Compatible with histological staining protocols, including H&E, Weigert’s, and Picrosirius red [70]. ➢ Compatible with immunofluorescence staining protocols [70]. ➢ Has been used in both whole animal and ex vivo tissue applications [70,101,102,103].	➢ Weak affinity for the arterial wall [101]. ➢ Has been reported to decalcify tissue [110]. ➢ It can diffuse into surrounding tissue in whole animal preparations [101]. ➢ Can cause tissue shrinkage [103,110].
Verhoeff’s stain (iodine, aluminum, and iron) [101]	➢ It can provide visualization of the aorta, carotid, renal, hepatic, and coronary arteries in situ by µCT [101]. ➢ Minimal diffusion into surrounding tissue in whole animal preparations [101].	➢ Has only been used in whole animal preparations [101].

**Table 3:** Overview of different sample preparation techniques for µCT.

Technique	Advantages	Limitations
Liquid immersion	➢ Liquids such as corn oil [73,94] offer a different density to water, providing a clearer definition of the soft arterial wall.	➢ Samples prone to physical damage. ➢ Samples prone to movement during scanning. ➢ Liquids may increase the noise in the image and impact beam hardening. ➢ Not suitable for long-term storage. ➢ May not be compatible with subsequent 2-D histological and immunohistochemical analysis.
Agarose gels	➢ Prevents sample movement during scanning.	➢ Samples prone to physical damage. ➢ Not suitable for long-term storage. ➢ Difficult to retrieve intact tissue from the gel after scanning.
Plastic resins	➢ Suitable for long-term storage. ➢ Prevents sample movement during scanning. ➢ Low tendency to form bubbles during polymerization. ➢ Molds can be used to create rounded resin blocks without edges.	➢ Not compatible with subsequent 2-D histological and immunohistochemical analysis.
Paraffin wax	➢ Samples prepared using standard histological procedures. ➢ Suitable for long-term storage. ➢ Prevents sample movement during scanning. ➢ Molds can be used to create rounded wax blocks without edges. ➢ Tissue samples can be re-embedded into larger paraffin wax blocks after scanning, enabling the tissue to be sectioned and stained.	➢ Tendency for bubbles or cracks to form in the wax. ➢ X-ray absorption coefficient of paraffin wax and biological tissue can be similar.

**Table 4:** Comparison of biochemical, histological, and µCT techniques.

	*o*-cresolphthalein	Histology	µCT
**Time**	1–2 days.	1–3 weeks to section, stain, and analyze a single blood vessel.	Up to 1 h to carry out a low-objective µCT scan of an intact blood vessel. Tissue features can be analyzed within a few hours.
**Processing**	Acidic digestion results in the destruction of the tissue, so it is unknown whether increased calcium content is representative of actual calcification or calcium excess.	Calcified deposits are prone to producing shearing artefacts and tissue damage during sectioning, which precludes the accurate quantification of calcification in blood vessels.	Intact blood vessels can be imaged in 2-D and 3-D.
**Analysis**			
(i) Quantification of calcification volume	Yes	Yes, but 2-D calcification volume can only be quantified in individual tissue sections.	Yes, 3-D calcification volume can be quantified in an intact, whole blood vessel.
(ii) Quantification of calcification load	No	No	Yes
(iii) Arterial medial vs. intimal calcification	No	Yes	Yes
(iv) Micro vs. macro-calcifications	No	Yes, but sectioning-induced artefacts often preclude the visualization and accurate quantification of micro vs. macro-calcifications.	Yes
(v) Calcification localization and distribution	No	Yes, but multiple serial tissue sections need to be cut, stained, imaged, and analyzed from a single blood vessel.	Yes, visualized in a whole blood vessel in 3-D.
(vi) Correlation with complementary analysis in the same arterial segment (e.g., extracellular matrix remodeling, IHC)	No	Yes, but sectioning-induced artefacts frequently preclude the accurate quantification of calcification.	Yes
(vii) Experimental bias	No	Yes, by selecting areas of analysis either randomly along the profile of the vessel, or at the site of maximum calcification.	No

## Abbreviations

ApoE	Apolipoprotein E
CKD	Chronic kidney disease
ECM	Extracellular matrix
LCM-MS	Laser capture microdissection coupled to mass spectrometry
LDLR	Low-density lipoprotein receptor
nCT	X-ray nano computed tomography
VSMC	Vascular smooth muscle cell
µCT	X-ray micro-computed tomography
µPET	Micro-positron emission tomography
